# The experiences of autistic doctors: a cross-sectional study

**DOI:** 10.3389/fpsyt.2023.1160994

**Published:** 2023-07-18

**Authors:** Sebastian C. K. Shaw, Alexander Fossi, Laura A. Carravallah, Kai Rabenstein, Wendy Ross, Mary Doherty

**Affiliations:** ^1^Department of Medical Education, Brighton and Sussex Medical School, Brighton, United Kingdom; ^2^Centre for Autism and Neurodiversity, Thomas Jefferson University, Philadelphia, PA, United States; ^3^Department of Pediatrics and Human Development and Department of Medicine, Michigan State University, East Lansing, MI, United States; ^4^East Sussex Healthcare NHS Trust, St. Leonards, United Kingdom; ^5^Department of Neuroscience, Brighton and Sussex Medical School, Brighton, United Kingdom

**Keywords:** autism, autistic, doctors, cross-sectional, survey, medical education, wellbeing

## Abstract

**Introduction:**

Medicine may select for autistic characteristics. As awareness and diagnosis of autism are growing, more medical students and doctors may be discovering they are autistic. No studies have explored the experiences of autistic doctors. This study aimed to fill that gap.

**Methods:**

This is a cross-sectional study. A participatory approach was used to identify the need for the project and to modify a pre-existing survey for use exploring the experiences of autistic doctors.

**Results:**

We received 225 responses. 64% had a formal diagnosis of autism. The mean age of receiving a formal diagnosis was 36 (range 3–61). Most were currently working as doctors (82%). The most common specialties were general practice / family medicine (31%), psychiatry (18%), and anesthesia (11%). Almost half of those working had completed specialty training (46%) and 40% were current trainees. 29% had not disclosed being autistic to anyone at work. 46% had requested adjustments in the workplace but of these, only half had them implemented.

Three quarters had considered suicide (77%), one quarter had attempted suicide (24%) and half had engaged in self-harm (49%). 80% reported having worked with another doctor they suspected was autistic, but only 22% reported having worked with another doctor they *knew* was autistic. Having never worked with a potentially autistic colleague was associated with having considered suicide.

Most preferred to be called “autistic doctors” (64%). Most considered autism to be a difference (83%). Considering autism to be a disorder was associated with preference for the term “doctors with autism,” and with having attempted suicide.

**Conclusion:**

Autistic doctors reported many challenges in the workplace. This may have contributed to a culture of nondisclosure. Mental health was poor with high rates of suicidal ideation, self-harm, and prior suicide attempts. Despite inhospitable environments, most were persevering and working successfully. Viewing autism as a disorder was associated with prior suicide attempts and a preference for person-first language. A neurodiversity-affirmative approach to autism may lead to a more positive self-identity and improved mental health. Furthermore, providing adequate supports and improving awareness of autistic medical professionals may promote inclusion in the medical workforce.

## 1. Introduction

Autism refers to a set of lifelong differences in how people communicate, interact, socialize, and behave (1, 2). Autistic people have individual strengths and challenges, which can include hyperfocus, differences in sensory perception, special interests, and anxiety (3). The estimated worldwide (and UK) prevalence of autism is at least 1% (1, 4, 5). More recently, one study in Northern Ireland has found a 4.7% prevalence in school aged children (6). The rate of diagnosis has increased steadily in recent years, which correlates with better awareness of autism, increased screening, and more accuracy in diagnosis (1). There is a growing understanding that there are geographic and demographic disparities in rates of diagnosis, with women, socio-economically disadvantaged populations, and those in countries with less awareness or more stigma around autism all being significantly less likely to receive a diagnosis (1, 7). In addition, we have an incomplete understanding of those who may not receive a diagnosis but who self-identify as autistic, and these individuals are unlikely to be recorded in the prevalence data. In this paper we have chosen to use identity-first language (“autistic person” rather than “person with autism”). This reflects the preferences of our autistic authors and current research on the topic, which finds that autistic people generally prefer identity-first language (8, 9). We recognize that some readers will disagree with this choice, and we wish to affirm the ways that autistic people choose to identify or refer to themselves.

Historically, research on autism has focused on deficits and difficulties. In recent years, there has been increasing acceptance of the neurodiversity paradigm, which challenges this pathologizing approach with a recognition that autism is not an inherent flaw or disordered way of being (10). Instead, aligned with the social model of disability, this paradigm scrutinizes social, cultural, political, and environmental factors as causes of contextual disability that traditionally fuelled sentiments of disorder (11, 12). Thus, autism may be thought of as a common set of differences. The benefits of the neurodiversity paradigm are significant, enabling us to have a conversation that does not focus on deficits, emphasizes the importance of diverse and neurodiverse communities, and takes a wider perspective, thinking about how external factors impact the capabilities and success of each individual (13, 14). While we can still identify autistic populations and characteristics, this framing allows us to do so while acknowledging the wealth of neurological diversity that exists in the population we are talking about, and the external influences of our social world (15).

Medicine may select for some common autistic characteristics, including attention to detail, pattern recognition, and a conscientious work ethic (2, 3). As awareness and diagnosis of autism are growing, more medical students and doctors may be discovering they are autistic, and this may occur at any stage through their training or working lives. Some reach diagnosis following difficulties in stressful clinical environments, or highly demanding career paths – and that support from employers, including occupational health and professional supervisors is inconsistent, with some colleagues refusing to believe a qualified doctor could be autistic (16). This may reflect the insidious nature of stereotypes. Stereotypes surrounding autism permeate all facets of society, including the medical workforce (17). In a symbiotic fashion, such stereotypes may be both born of and act to reinforce the tragedy narrative surrounding autistic people (18). Being autistic in such an environment may foster internalized ableism and may promote a lack of disclosure. For example, a recent study found that only 63% of autistic adults in employment had disclosed being autistic (19). This may be of particular importance within the medical workforce, where we know that disclosure of disability more generally is scarce, through fear of being seen to show weakness (20, 21). In fact, in a recent sample of 6,000 American physicians, those with self-reported disabilities were significantly more likely to be abused both by co-workers and patients, including actual physical harm (22).

In recent years, we have seen a near-exponential rise in recognition of intersectionality and diversification within the medical workforce. This has been particularly evident within the UK, where both the General Medical Council and the Medical Schools Council have produced supportive guidance around diversity and inclusion (23, 24). Such guidance reinforces the rights and needs of autistic people in relation to reasonable adjustments to their education and work. In the UK, this is also mandated by law (25). In addition to matters of social justice, such guidance is also driven by the recognition of the fact that “a diverse population is better served by a diverse workforce that has had similar experiences and understands their needs” (23). Despite such positive steps, evidence suggests we may still have a long way to go in our drive for a truly inclusive workforce (26). A commitment to such change must set its sights on the longer term and must also consider cultural and systemic factors at all levels of medical education and training. The actual study of medicine is as much cultural as it is factual (27). Throughout their medical studies, students and trainees undergo a transition into this culture, finding their own place within the vast medical world, alongside its associated language and practices. Previous research has found that neurodivergent people may struggle during training, through experiences of bullying, othering, and falling victim of the competitive system (28–30). Such experiences have also been reported specifically by autistic medical students (31). To that end, improving the experiences of autistic (and of otherwise neurodivergent) people within the medical workforce is vital. However, no published research has explored the experiences of autistic doctors. Our overarching aim was, therefore, to explore the experiences of autistic clinicians and the benefits of participation in a supportive community of those with similar experiences. Here, we present an analysis of our quantitative findings. We intend to analyze and publish our qualitative data elsewhere.

## 2. Materials and methods

### 2.1. Study type

This is a cross-sectional study in the form of an online survey.

### 2.2. Ethics

This study was approved by the Health Service Executive North East Area Research Ethics Committee.

### 2.3. Study context

Autistic Doctors International is an online support group, which was founded by MD in 2019. Membership requires a medical degree (or to be in the last year of medical school) and either a formal diagnosis or self-identification as autistic. At the time of conducting this study, the group had over 500 members.

### 2.4. Survey design

The survey was adapted, with permission, from the Autistic School Staff survey (32), which had been developed in conjunction with a committee of three autistic school staff to explore the experiences of autistic school staff based in the UK. A team of five autistic doctors adapted the survey to an international medical context suitable for distribution to members of Autistic Doctors International. Changes to the survey included adapting questions to the medical field, adding several questions focused on medical school experience, specialty selection, and medical training experiences, and the addition of a section asking about participants’ experiences with Autistic Doctors International.

The final survey included a mixture of open and closed questions along with Likert scale and multiple-choice questions. Free text boxes were included throughout. The survey consisted of 32 core questions, with additional questions contingent on participant responses. Including the contingent questions, there were 121 possible questions included.

### 2.5. Piloting and refinement

The survey was initially piloted with a small group of autistic doctors (*n* = 7). Following this, the survey was further refined. For example, consideration of gender was switched to a free text box. Other refinements included reorganization of sections to reduce redundant questions, improvements to the wording and language of some questions, and the addition of two free text boxes allowing users to clarify other responses. No core questions were added or removed at this stage, but the total number of possible questions was increased from 121 to 123.

### 2.6. Recruitment and data collection

This study was open to all members of Autistic Doctors International. Study invitations containing links to the online survey were shared with all members via Facebook and WhatsApp April–July 2022. The survey began with a participant information sheet. Following this, informed consent was received prior to progressing to the questionnaire.

### 2.7. Data sorting

Once data collection was complete, SS and AF individually reviewed the dataset. The wider research team then met to agree protocols for data sorting, such as those for converting qualitative responses about gender and countries into statistically analyzable formats. Responses to free-text questions on race/ethnicity, countries where training was received, time in practice, gender, level of training, and medical specialty were categorized.

### 2.8. Data analysis

Analyses were conducted by SS using Statistical Package for the Social Sciences (SPSS) 28. Descriptive statistics were used to explore frequencies. Chi-square was used to test for associations between categorical variables. Linear-by-linear association was also checked where appropriate. Significance was assessed at the *p* < 0.05 level.

## 3. Results

The survey received 225 responses. Whilst Autistic Doctors International member levels are fluid in nature, and spread across various platforms, this represents an approximately 40% response rate. Demographic details are outlined in Table 1.

**Table 1:** Demographic information.

	**%**	**N**
**Current age**		
20–29	12.9	29
30–39	36.2	81
40–49	29.5	66
50–59	14.3	32
60–69	5.4	12
70 or over	1.8	4
Total N =		224
**Gender identity**		
Female	81.3	178
Male	11.9	26
Non-binary, agender, or gender fluid	6.8	15
Total N =		219
**Sexual orientation**		
Straight	57.6	129
Bisexual	11.2	25
Asexual	8.5	19
Do not know	7.1	16
Gay/lesbian	5.8	13
Pansexual	4.5	10
Not listed	3.1	7
Prefer not to say	2.2	5
Total N =		224

Respondents resided in a variety of countries (Table 2). Most (61%) were living in the United Kingdom, followed by Australia (27%), the United States (16%), and Canada (11%).

**Table 2:** Current geographical locations.

**Location**	**%**	**N**
United Kingdom	61	136
Australia	12.1	27
United States	7.2	16
Canada	4.9	11
Ireland (Republic)	4	9
New Zealand	1.8	4
France	1.3	3
Romania	1.3	3
Denmark	0.9	2
Germany	0.9	2
South Africa	0.9	2
Austria	0.4	1
Brazil	0.4	1
Finland	0.4	1
Malaysia	0.4	1
Malta	0.4	1
Norway	0.4	1
Spain	0.4	1
Sweden	0.4	1
Total N =		223

### 3.1. Diagnosis

Most (64%, *n* = 143) had a formal diagnosis of autism. The rest self-identified as autistic. Of those with a formal diagnosis, the mean age of receiving this was 36 (range 3–61). A few were diagnosed before the age of 18 (6%, *n* = 8).

One third (34%, *n* = 74) first suspected they were autistic when they were junior doctors/residents, and nearly one third first suspected when they were consultants/attendings or general practitioners (30%, *n* = 65). Some first suspected before they ever went to medical school (14%, *n* = 30), and a few first suspected whilst they were medical students (11%, *n* = 23).

### 3.2. Co-occurring conditions

Over a third had been diagnosed with generalized anxiety disorder (40%, *n* = 91) or depression (39%, *n* = 87). Nearly one third had also been diagnosed with ADD/ADHD (29%, *n* = 66). Almost one fifth had been diagnosed with post-traumatic stress disorder (18%, *n* = 40), over one tenth had been diagnosed with an eating disorder (13%, *n* = 30), and one tenth had been diagnosed with social anxiety disorder (10%, *n* = 23).

### 3.3. Medical school experiences

Most did not know or suspect they were autistic when at medical school (74%, *n* = 164). Of those who knew they were autistic, most did not disclose this to their medical school (72%, *n* = 43). Of those who did disclose, only half received adjustments (53%, *n* = 9). A quarter reported medical school taking them longer to complete than their peers (25%, *n* = 57). Of these, almost all felt being autistic played a part in that (89%, *n* = 50).

### 3.4. Current employment

Most were currently working as doctors (82%, *n* = 183). Some had previously worked as doctors but were not currently practicing (14%, *n* = 32), and a few had never worked as doctors after graduating from medical school (4%, *n* = 9).

At the time of completing the survey, almost half were consultants/attendings or general practitioners (46%, *n* = 84). Two fifths were currently trainees/junior doctors/residents (40%, *n* = 73). Some were non-training grade associate specialists (11%, *n* = 20) and a few reported other roles/routes (3%, *n* = 5). Of those with a childhood diagnosis (*n* = 8), seven were currently working as doctors with one yet to start.

Half were employed in a permanent position (51%, *n* = 113) and a quarter in a temporary position (27%, *n* = 59). A tenth were not currently employed (11%, *n* = 25), and a tenth were self-employed (11%, *n* = 24). Of those working, half were working fulltime (50%, *n* = 96) and half were working parttime (50%, *n* = 97).

One third were in general practice/family practice (32%, *n* = 67), nearly a fifth were in psychiatry (18%, *n* = 37), and a tenth were in anesthetics (11%, *n* = 24). A few were in internal medicine (6%, *n* = 13), pathology (4%, *n* = 8), or surgery (3%, *n* = 6). A range of other specialties were represented in smaller numbers, which were collected into an “other specialty” category (26%, *n* = 54).

Overall, three quarters usually enjoyed their work as doctors (74%, *n* = 158).

### 3.5. Autistic perspectives

Three quarters felt that being autistic helped them in their work as doctors (73%, *n* = 133). Three quarters also felt that being autistic hindered their work as doctors (73%, *n* = 131). Three quarters reported executive functioning challenges at work (77%, *n* = 162) and three quarters reported sensory issues being challenging at work (75%, *n* = 156).

Most preferred to be called “autistic doctors” (64%, *n* = 145), with less than a fifth preferring to be called “doctors with autism” (18%, *n* = 40). Most considered autism to be a difference (83%, *n* = 187), half considered it to be an identity (54%, *n* = 122), half considered it to be a disability (52%, *n* = 118), and only just over a tenth considered it to be a disorder (13%, *n* = 30). Considering autism to be a disorder was significantly associated with preference for the term “doctors with autism” (*p* < 0.001).

Four fifths reported having worked with another doctor they suspected was autistic at some stage in their career (80%, *n* = 168), but only one fifth reported having worked with another doctor they *knew* was autistic (22%, *n* = 46).

### 3.6. Disclosure at work

One third had disclosed being autistic to their supervisor/consultant (32%, *n* = 73), nearly a third had disclosed to their colleagues (30%, *n* = 68). One fifth had disclosed to occupational health (19%, *n* = 44) and nearly a fifth had disclosed to human resources (16%, *n* = 35). Nearly a tenth had disclosed being autistic to their patients (8%, *n* = 19). Nearly a third had disclosed to *no one* at work (29%, *n* = 65).

### 3.7. Reasonable adjustments at work

Less than half had requested adjustments in the workplace (46%, *n* = 98). Of those who had, only half had them implemented (49%, *n* = 48). Three quarters of those who received adjustments felt they were helpful in their jobs as doctors (75%, *n* = 36). Table 3 outlines changes that respondents felt would improve their ability to do their jobs.

**Table 3:** Changes felt to make their jobs easier/better.

**Desired adjustment**	**%**	**N**
A more manageable workload	50	112
Being able to be open about being autistic	49	111
A better work environment (e.g., with less noise and different lighting)	48	109
More autism understanding from colleagues	44	98
More flexibility from employers	44	98
More autism understanding from employers	42	94

### 3.8. Troubles at work

A quarter reported issues at work that had involved human resources or a disciplinary process (24%, *n* = 50). A tenth reported issues at work that had resulted in a formal regulatory/licensing process (9%, *n* = 20). Nearly a third reported issues at work requiring union or legal representation (30%, *n* = 63). While only 21% (*n* = 47) reported challenges in communication with patients, most reported challenges from the judgement or attitudes of other people at work (65%, *n* = 137). Approximately three-quarters experienced challenges in communication with peers (76%, *n* = 161), supervisors (74%, *n* = 155) and management (75%, *n* = 157).

### 3.9. Mental health

Half of our respondents had engaged in self-harm (49%, *n* = 106), three quarters had considered suicide (77%, *n* = 166), and one quarter had previously attempted suicide (24%, *n* = 40). Having engaged in self-harm was significantly associated with having considered suicide (*p* < 0.001) and having attempted suicide (*p* < 0.001). Considering autism to be a disorder was associated with having attempted suicide (*p* = 0.019, 43 vs. 21%).

Gender was associated with having engaged in self-harm (*p* < 0.001). Self-harm was reported by 73% of those identifying as non-binary, agender or genderfluid, 51% of those identifying as female, and 17% of those identifying as male. Having a formal autism diagnosis was also associated with having engaged in self-harm (*p* < 0.001). In addition, sexual orientation was also significantly associated with having engaged in self-harm (*p* = 0.007): pansexual (30%), straight (39%), bisexual (63%), gay/lesbian (69%), do not know (79%), not listed (86%).

Having requested adjustments at work was associated with having engaged in self-harm (*p* = 0.039), with only 42% of those not requesting adjustments having engaged in self-harm, compared with 50% of those who received adjustments and 64% of those who requested adjustments but did not receive them. Current grade was also associated with having engaged in self-harm (*p* = 0.003). Self-harm was reported by 64% of junior doctors/residents, 21% of non-training grade associate specialists, and 42% of consultants/attendings or general practitioners having completed training/been board certified (*p* = 0.022). The stage at which respondents first suspected they might be autistic was also associated with having engaged in self-harm (*p* = 0.01). Self-harm was reported by 77% of those who first suspected they might be autistic before medical school, 50% of those who first suspected at medical school, 46% of those who first suspected as junior doctors / residents, and 41% of those who first suspected as consultants/attendings or general practitioners having completed training / been board certified (*p* = 0.023).

Whether respondents were still working as doctors was associated with having *attempted* suicide (*p* = 0.002). Having attempted suicide was reported by 22% of those currently working as doctors, 75% of those having never worked as doctors, and 20% of those having previously worked as doctors but who were not currently practicing. Having never worked with another doctor who they suspected to be autistic was significantly associated with having *considered* suicide (*p* = 0.022, 90% vs. 73%).

There was an association between having previously considered suicide and respondents’ views on whether Autistic Doctors International had been positive for their mental health (*p* = 0.017): strongly agreed it was positive (84%), agreed (67%), neither agreed nor disagreed (86%), disagreed (50%) (*p* = 0.004).

Having engaged in self-harm was associated with having disclosed being autistic to their consultant/supervisor (*p* = 0.002) and having disclosed to occupational health (*p* < 0.001). Having *not* disclosed being autistic to patients was associated with having engaged in self-harm (*p* = 0.017) and having attempted suicide (*p* = 0.043). Having disclosed being autistic to *no one* at work was associated with having never engaged in self-harm (*p* < 0.001) and having never considered suicide (*p* = 0.011).

### 3.10. Autistic doctors international

Most respondents found their membership personally beneficial (88%, *n* = 196). Almost all felt membership of the group was beneficial for autistic doctors in general (96%, *n* = 216). Three quarters felt membership had been positive for their mental health (72%, *n* = 162).

## 4. Discussion

Our findings tell the story of a diverse group of autistic doctors across the world who in many ways are thriving despite numerous barriers. Many discovered their autistic identity later in life, but few felt comfortable disclosing this either in medical school or to future peers and employers. Disclosure to colleagues, supervisors, and human resources was uncommon, even though half of respondents reported that being able to be more open about autism would improve their work experience. This suggests the luxury of disclosure may be unavailable, excessively burdensome, or even harmful for many autistic doctors, despite the potential benefits that being open about their autistic status might bring. This mirrors the wider literature, which suggests that, whilst disclosure can be an appealing option for autistic employees, it is difficult to access in many workplaces and is often associated with stigma and discrimination from supervisors and co-workers (33). Our findings support previous calls for defined disclosure processes, supported by workplace to address stigma and provide accommodations (34, 35).

In our sample, half had engaged in self-harming behavior and over three quarters had considered suicide, compared with lifetime suicidal ideation rates of under 10% in the general population (36). Comparatively, the prevalence of suicidal ideation in the wider autistic population is also lower, sitting between 19.7 and 66% (37). Similarly, suicidal ideation in medical doctors, whilst well recognized as higher than the wider population, is also much lower – reported to be between 6.3 and 24.8% (38). It is therefore imperative to consider why the prevalence of suicidal thoughts may have been higher within our sample of autistic doctors. While we cannot draw a straight line that connects difficulties at work with high levels of self-harm and suicidal ideation, it is notable that, among those who requested adjustments in our sample, rates of self-harm were higher. This was particularly true for those who requested adjustments and did not receive them. We do see a pattern of those who did not disclose being less vulnerable to self-harm and suicidal ideation. This suggests the possibility that those who face more significant needs for support and accommodation are both less able to avoid disclosure and more likely to experience mental health difficulties. Whichever causality one chooses to follow, the process of disclosure itself must be accessible and positive. A world where doctors who seek adjustments are less likely to work and more likely to self-harm is not one that is helping autistic doctors to be healthy and productive contributors to the medical profession – potentially increasing health inequity through weeding out the benefits that autistic doctors bring to the profession for autistic patients (23). Furthermore, our finding that having never worked with another doctor they suspected to be autistic was associated with having considered suicide is important. Whilst this is an association and not a causation, this may provide some preliminary evidence to support the assertion that openly autistic role models play a key role within medical education and the wider medical workforce. As previously argued, “witnessing colleagues with whom we can identify and being able to learn from their successes and struggles may make the difference between leaving a career we dreamed of, or pursuing it, more aware of our strengths, our vulnerabilities and the right to advocate for accommodations” (18).

In this same vein, we see that those who found membership in Autistic Doctors International to be most positive were also more likely to have considered suicide. Given that there is clearly a significant set of barriers to disclosure for people with any level of adjustment needs, it is possible that these individuals might find membership in a group where they can comfortably disclose to be a positive experience. While Autistic Doctors International is unable to directly create adjustments in the workplace, it can be a way to find peer support and a welcoming community – exactly those things that may be lacking in a workplace with a culture of nondisclosure. Factors contributing to autistic people succeeding at work include peer support, provision of mentorship, and supportive communication (especially through an indirect platform, such as virtually) (39–42). Ideally, these positive factors would already exist in the medical workplace, but in this study, we see that many do not have access to these types of positive supports formally. Organizations like Autistic Doctors International, therefore, provide access to a supportive peer group and may help ameliorate any fear/damage introduced by workplaces that are not prepared to properly accommodate and embrace their autistic employees. This is in keeping with the wider literature, which shows that social support can be protective against emotional distress and burnout in medical students and doctors (43, 44). In the wider population, it has also been found to protect against suicide (45).

One result that is deserving of attention is that, among the relatively low number of respondents who disclosed their autistic status to patients, rates of self-harm were significantly lower. This is an area worth additional exploration. The wider literature suggests that disclosure of personal mental ill health or disability to patients is culturally frowned upon within medicine (46). Within our findings, it is not possible to assert causation here. One the one hand, respondents who experienced better mental health may feel more confident in disclosing to patients for patient benefit. On the other, could it be possible that a sense of camaraderie or acceptance with autistic patients in some way fostered improved mental health in our respondents? From another perspective, evidence suggests that autistic people are more comfortable in seeing a doctor whom they *know* to be autistic. In fact, one of the most common requests received by Autistic Doctors International is from autistic people asking for recommendations of openly autistic doctors they could approach for healthcare. Considering factors that may facilitate positive disclosure with patients, a better understanding of what makes these providers feel comfortable enough in their environment to disclose could help us support the creation of workplace environments that are more positive for the many other autistic doctors who do not feel similarly comfortable in disclosing. It is worth examining what is different about these workplaces – is it the patient population, a workplace culture of disclosure, a particular specialty that is more welcoming, or are there other factors that we can identify to create a set of best practices in supporting disclosure and mental health among autistic doctors?

Our respondents’ preference for identity-first language and framing of autism as *difference* rather than disorder is in keeping with the wider autistic community perspective (9). One of our most striking findings was the association between the conceptualization of autism as disorder, the preference for person-first language and worse mental health as evidenced by previous suicide attempts. A growing awareness of the harm associated with a stigmatizing, deficit-based perspective on autism is leading to a re-framing towards a neurodiversity-affirmative approach, which may offer benefits in terms of mental health (47, 48). In our findings, we see the need for this approach to be adopted not just in patient care, but in how we think about employment in the medical profession. Most did not feel comfortable enough to disclose being autistic to others in the workplace. As a result, these people are consistently camouflaging or masking their autistic status, putting on a non-autistic façade for others in professional settings. Such practice takes a continual effort on the part of autistic people, and we know that masking can contribute to burnout, discontinuing employment, and even serious mental health issues and suicide (49, 50) – many of which are major issues within the medical workforce to begin with (51). To that end, suppressing one’s autistic identity in this way may be deeply harmful. In the words of Maya Angelou, “there is no greater agony than bearing an untold story inside you” (52). To try and separate autistic doctors from ‘their autism’ is an impossible task, and yet this is essentially what we are asking them to do when we provide workplaces that cannot offer basic accommodations or make disclosure a realistic and supported option.

### 4.1. Strengths and limitations

This is the first study to explore the experiences of autistic doctors. It benefits from a participatory approach, whereby it was conceptualized and driven largely by autistic doctors. This allowed deeper insight into the needs and experience when designing the survey. The survey also benefited from being originally based on a previously developed study of autistic school staff (32).

During the analysis stage, we were informed that two respondents may or may not have completed the survey twice. We were, however, unable to confidently locate these potential duplicates. This is a limitation. The 14% (*n* = 32) who had previously worked as doctors but were not currently working may have stopped practicing at retirement age or may have ceased practice prematurely due to burnout or other reasons. Our survey did not discriminate between these groups, which is a further limitation. When interpreting our findings, it is also worth considering that we did not attempt statistical validation of our survey during its construction. It is also worth considering the limits associated with self-report data, and the fact that cross-sectional data cannot infer causation within associations.

Furthermore, it is important to consider our results in the context of our recruited sample. All participants were members of Autistic Doctors International at the time of completing the survey. The peer support focus of this group may impact the experiences reported. It is also worth noting that our survey was conducted online and in English. As such, this would have limited participation from those who do not read/write in English and from those without internet access.

Finally, it is vital to consider the potential implications of our findings themselves, given their striking nature – in particular, the preference for considering autism a difference, and the association between considering autism a disorder and higher rates of reported suicide attempts. As aforementioned, this insight holds the potential to influence positive change through supporting the adoption of neurodiversity-affirmative practices and identity-first language. Such impacts may include improved mental health and optimum outcomes for the autistic community more widely (47, 53). This is in line with our own philosophical positioning as the authors. However, also in line with our positioning, it is important that these findings around ‘difference’ are not mistaken as a dismissal of support needs or disability. The neurodiversity paradigm does not refute disablement, and indeed over half of our respondents identified as disabled. Instead, this paradigm shifts the focus of causation to external factors such as social, cultural, historical, political, and environmental causes (12, 47). To that end, we believe that a truly neurodiversity-affirmative approach should embrace difference through its aim to ameliorate such disablement, achieved through active inclusion and consideration of potentially ableist approaches – both on the ground and at higher, systems levels. From a reflective perspective, we feel this clarification is important to protect against potential misunderstanding of our findings. Otherwise, this risks the weaponization of heterogeneity being targeted against the autistic community, whereby a difference perspective might be mistaken as, or portrayed as, refuting disablement, thus creating a community fracture, and seemingly supporting functioning labels – i.e., the postulation that some of us are different and others are disabled. This risks either the downplay of support needs or the downplay of agency. Contrary to this, however, the high rates of mental ill health identified within this study do indeed evidence the importance of actively adjusting our educational and workplace systems within medicine to support autistic doctors who otherwise face a very real disablement.

## 5. Conclusion

Autistic doctors reported many challenges in the workplace. This may have contributed to a culture of nondisclosure. Mental health was poor with high rates of suicidal ideation, self-harm, and prior suicide attempts. Despite inhospitable environments, most were persevering and working successfully. Viewing autism as a disorder was associated with prior suicide attempts and a preference for person-first language.

A neurodiversity-affirmative approach to autism may lead to a more positive self-identity and improved mental health. Furthermore, providing adequate supports and improving employer and peer awareness of autistic medical professionals may promote inclusion in the medical workforce. Employing a well-supported and neurodiverse set of medical professionals will mean that the diversity of the public is reflected in their medical providers, with likely improved patient outcomes. This is an area requiring further research to optimize the circumstances that lead to happier lives and more productive medical careers for this group.

*“There comes a point where we need to stop just pulling people out of the river. We need to go upstream and find out why they’re falling in.”* (Demond Tutu).

## Data availability statement

The raw data supporting the conclusions of this article will be made available by the authors, without undue reservation.

## Ethics statement

The studies involving human participants were reviewed and approved by the Health Service Executive North East Area Research Ethics Committee. The patients/participants provided their written informed consent to participate in this study.

## Author contributions

MD, LC, KR, AF, and WR: conception and design. AF, MD, LC, KR, WR, and SS: data collection, drafting manuscript, and agreeing final submission. SS: data analysis. SS, AF, MD, and LC: interpretation of results. All authors contributed to the article and approved the submitted version.

## Funding

We are grateful to Jeanette and Chris Alwine for providing funding support. We are also grateful to Thomas Jefferson University, who provided support for the publication fees via the Thomas Jefferson University Open Access Fund.

## Conflict of interest

SS, LC, KR, and MD are all leading members of Autistic Doctors International.

The remaining authors declare that the research was conducted in the absence of any commercial or financial relationships that could be construed as a potential conflict of interest.

## Publisher’s note

All claims expressed in this article are solely those of the authors and do not necessarily represent those of their affiliated organizations, or those of the publisher, the editors and the reviewers. Any product that may be evaluated in this article, or claim that may be made by its manufacturer, is not guaranteed or endorsed by the publisher.
